# Successful treatment of necrobiotic xanthogranuloma with subcutaneous immunoglobulin and topical roxulitinib

**DOI:** 10.1016/j.jdcr.2026.06.011

**Published:** 2026-06-15

**Authors:** Hanni Robinson, Gil Armoni-Weiss, Stephanie Benshushan, Vered Molho-Pessach

**Affiliations:** a Department of Dermatology, The Faculty of Medicine, Hebrew University of Jerusalem, Hadassah Medical Center, Jerusalem, Israel; b Department of Pathology, The Faculty of Medicine, Hebrew University of Jerusalem, Hadassah Medical Center, Jerusalem, Israel

**Keywords:** monoclonal gammopathy, necrobiotic xanthogranuloma, non-Langerhans cell histiocytosis, subcutaneous immunoglobulin (HyQvia), topical roxulitinib

## Introduction

Necrobiotic xanthogranuloma (NXG) is a rare, non-Langerhans cell histiocytosis, predominantly affecting individuals in their sixth decade of life. It is characterized by indurated yellowish nodules and plaques, with occasional ulceration and predilection for the periorbital region, though lesions may occur on the trunk and extremities. Paraproteinemia is present in approximately 80% of cases, typically manifesting as IgG kappa or lambda monoclonal gammopathy due to plasma cell dyscrasia or a B cell lymphoproliferative disorder.^1^^,^^2^

The pathogenesis of NXG remains incompletely understood, though paraproteins, and especially the M protein, are hypothesized to trigger inflammatory cascades leading to granuloma formation.^2^, ^3^, ^4^ Recent genetic studies have implicated disruptions in cholesterol metabolism in a subset of patients without monoclonal gammopathy.^4^

Treatment of NXG remains challenging due to the rarity of the condition and lack of controlled trials. Reported therapeutic approaches include intralesional and systemic corticosteroids, intravenous immunoglobulin (IVIG), plasmapheresis, antimalarials, immunomodulators, and chemotherapy.^2^ We present a case of NXG successfully treated with subcutaneous immunoglobulin (SCIG, HyQvia), in combination with topical roxulitinib, representing a novel therapeutic approach for this challenging condition.

## Case report

A 51-year-old woman presented with a 2-year history of progressive, asymptomatic skin lesions affecting the trunk and extremities. Her past medical history was significant for iron-deficiency anemia, borderline antiphospholipid syndrome, and von Willebrand factor deficiency.

Physical examination revealed indurated yellowish-erythematous plaques ranging from 1 to 8 cm in diameter, over her chest, right flank, back, lower extremities and temporal aspect of her left forehead, with central ulceration in some of the plaques (Fig 1, *A*). Dermoscopy showed amorphous whitish structures, telangiectasias, and ulceration on a yellow background (Fig 2). General physical examination and an ophthalmologic evaluation were unremarkable.

**Fig 1:**
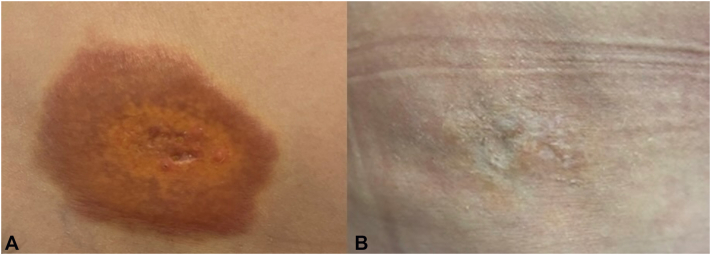
**A,** An indurated yellowish-erythematous plaque with central ulceration on the right flank, measuring approximately 6 cm in diameter. **B,** Hyperpigmented patch over right flank with a central scar after 1 year of treatment with SCIG and topical roxulitinib. *SCIG*, Subcutaneous immunoglobulin.

**Fig 2:**
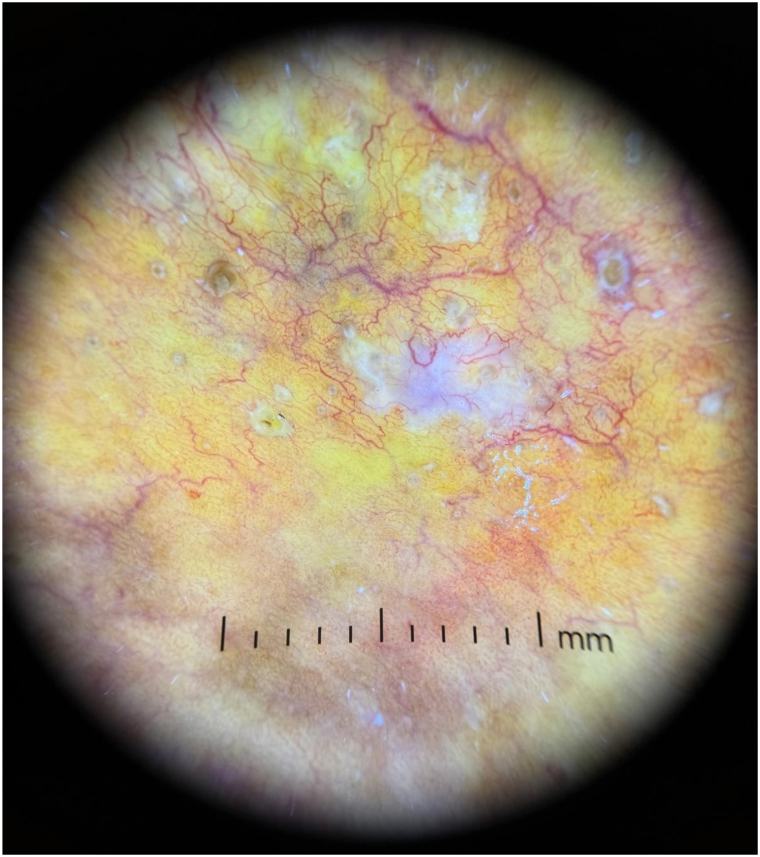
Dermoscopy demonstrating amorphous whitish structures, telangiectasias, and central ulceration on a yellow background.

Histopathological examination revealed alternating layers of degenerated collagen in the dermis with an inflammatory infiltrate composed of CD68-positive histiocytes, some demonstrating xanthomatization, and multinucleated giant cells including Touton-type giant cells. Cholesterol clefts were identified within the granulomatous inflammation (Fig 3).

**Fig 3:**
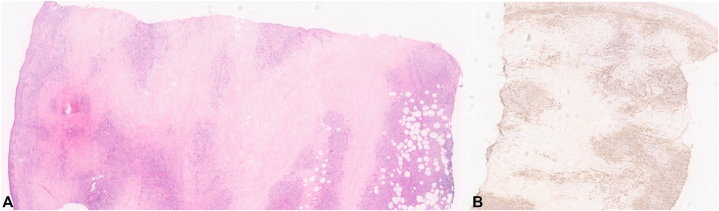
Histopathological findings from 3-mm punch biopsy (hematoxylin and eosin stain, original magnification ×25). **A,** In the dermis alternating layers of degenerated collagen with granulomatous inflammation composed of histiocytes, xanthomatized cells, and multinucleated giant cells including Touton-type giant cells. Cholesterol clefts are present within the inflammatory infiltrate. **B,** CD68 stains for histiocytes are positive.

Extensive laboratory evaluation revealed positive antinuclear antibodies at 1:40 titer. Serum protein electrophoresis and immunoelectrophoresis demonstrated an IgG lambda monoclonal protein. Additional immunologic abnormalities included elevated anti-β2-glycoprotein IgM antibodies (16.3 U/mL; normal 0-8 U/mL), elevated anti-cardiolipin IgM antibodies (35.9 U/mL; normal 0-7 U/mL), decreased complement C4 (5.6 mg/dL; normal 10-40 mg/dL), and positive cryoglobulin (2% in serum). Whole-exome sequencing of peripheral blood DNA did not reveal mutations in *ABCG5* or *ABCG8* genes.

Bone marrow biopsy demonstrated trilineage hematopoiesis with normo-cellularity for age (50%) and a mild increase in lambda-type plasma cells, consistent with monoclonal gammopathy of undetermined significance. Whole-body computed tomography scan showed in the liver, several hypodense lesions up to 1.2 cm in size, with peripheral nodular enhancement, suggestive of xanthogranulomas. This interpretation was based on their radiologic pattern in the context of histologically confirmed NXG. Transthoracic echocardiography was within normal limits, ruling out cardiac involvement. The combination of the clinical, histopathological, and hematologic findings was consistent with NXG.

Initial management consisted of oral prednisone 40 mg daily combined with topical clobetasol propionate 0.05% ointment for approximately 8 weeks, followed by 2 treatment sessions with intralesional steroids (Kenalog – triamcinolone acetonide) directed to the lesion sites, yielded only temporal improvement. Hence, a single infusion of IVIG was initiated, as well as topical roxulitinib 1.5% cream applied once daily.

Following the first IVIG infusion, the patient developed elevation of liver transaminases and dyspnea. SCIG therapy was proposed as a potential alternative.

The patient was initiated on HyQvia (Shire US Inc), at a dose of 60 grams administered subcutaneously every 2 weeks, with tapering and cessation of prednisone treatment. HyQvia is supplied as a ready-to-use solution of immune globulin 10% (100 mg/mL) co-formulated with recombinant human hyaluronidase, administered without solution. The full dose of 600 mL was delivered as a single subcutaneous injection at 1 abdominal site per session, facilitated by the hyaluronidase enabling large-volume administration at a single subcutaneous site. The SCIG administrations were well-tolerated without adverse effects.

Clinical improvement was noted after about 2 months following the initiation of SCIG therapy in conjunction with topical roxulitinib. A year after initiation of treatment, the patient has achieved near-complete clearance of the lesions across all affected sites (chest, right flank, back, lower extremities, and temporal forehead), with all treated lesions demonstrating a similarly favorable response, with residual hyperpigmented patches as the predominant findings (Fig 1, *B*). Focal cutaneous atrophy was also observed at treatment sites, which may represent an adverse effect of the use of topical or intralesional steroids, due to the hyaluronidase component of HyQvia, or may reflect tissue remodeling secondary to resolution of the underlying xanthogranulomatous infiltrate. Hepatic lesions remained stable on imaging. The patient continues hematological follow-up.

## Discussion

To the best of our knowledge, this case is the first to report successful treatment of NXG with SCIG and topical roxulitinib and highlights this combination as a viable therapeutic option.

IVIG has emerged as an effective treatment option for NXG, with multiple case reports and small series demonstrating clinical efficacy.^2^ IVIG’s mechanism of action mechanism in NXG is not well established but is believed to involve immunomodulatory effects on the paraprotein-mediated granulomatous inflammatory response. IVIG is thought to exert anti-inflammatory effects through modulation of Fc receptor expression and function, reducing proinflammatory cytokine release.^5^^,^^6^ IVIG therapy is associated with well-recognized adverse effects including headache, fever, nausea, thromboembolism, renal dysfunction, and hepatotoxicity.^5^

SCIG (HyQvia) combines immune globulin 10% with recombinant human hyaluronidase, an enzyme that temporarily increases subcutaneous tissue permeability by hydrolyzing hyaluronan, thereby enabling larger volume infusions at a single subcutaneous site. The hyaluronidase effect is reversible, with tissue architecture restored within 24-48 hours. The subcutaneous route is associated with less systemic adverse reactions, eliminating the risk of rapid infusion-related complications compared to IVIG.^7^

Prior to this report, only 2 reports have described the use of SCIG for dermatologic conditions. Speth *et al* reported 5 patients with refractory juvenile dermatomyositis, treated successfully with high-dose SCIG. Radin *et al* described clinical improvement in a patient with refractory pemphigus vulgaris.^8^^,^^9^ To our knowledge, the present case represents the first reported use of SCIG in NXG and more broadly, in non-Langerhans cell histiocytosis.

Our patient's sustained improvement with SCIG, despite concomitant corticosteroid tapering, suggests that SCIG may maintain therapeutic efficacy comparable to the intravenous formulation in this condition.

The addition of topical ruxolitinib, a JAK1/JAK2 inhibitor, as adjunctive therapy in this case is noteworthy. Only 1 prior case report has described topical JAK inhibitor use in NXG (tofacitinib)^10^ the anti-inflammatory effects of JAK inhibition may provide additional benefit by suppressing local cytokine signaling and macrophage activation. The relative contributions of systemic immunoglobulin and topical JAK inhibition, to our patient's improvement cannot be definitively determined.

The present case expands the recognized clinical spectrum of NXG. While periorbital involvement remains the hallmark, and rare reports have documented mucosal and laryngeal disease,^11^ the current case underscores that NXG can affect anatomically distinct sites in the skin and visceral organs. Management must be individualized according to lesion behavior, organ involvement, and treatment tolerance.

This case report emphasizes the importance in development of a standard management protocol for NXG. We acknowledge the limitations in our report including the absence of a control group and the use of combination therapy, which precludes definitive attribution of therapeutic benefit solely to SCIG, as well as uncertainty regarding the optimal dosing, frequency, and duration of therapy. Future studies are required to have a better understating of the disease and its treatment. In summary, this case demonstrates the feasibility of SCIG as an alternative immunoglobulin delivery route in NXG, particularly when intravenous administration is not tolerated. The therapeutic contributions of individual agents in this combination regimen cannot be disentangled, and our findings should be interpreted as hypothesis-generating rather than confirmatory of efficacy.

### Declaration of generative AI and AI-assisted technologies in the writing process

During the preparation of this work, the authors used Claude.AI in order to check for grammar and spelling mistakes. After using this tool, the authors reviewed and edited the content as needed and take full responsibility for the content of the published article.

## Conflicts of interest

None disclosed.
